# COVID-19 clinical presentation, management, and epidemiology: a concise compendium

**DOI:** 10.3389/fpubh.2025.1498445

**Published:** 2025-01-31

**Authors:** David P. Maison, Hawi Tasissa, Amelia Deitchman, Michael J. Peluso, Youping Deng, F. DeWolfe Miller, Timothy J. Henrich, Mariana Gerschenson

**Affiliations:** ^1^Department of Cell and Molecular Biology, John A. Burns School of Medicine, University of Hawaii at Manoa, Honolulu, HI, United States; ^2^Division of Experimental Medicine, Department of Medicine, University of California, San Francisco, San Francisco, CA, United States; ^3^Department of Clinical Pharmacy, University of California, San Francisco, San Francisco, CA, United States; ^4^Division of HIV, Infectious Diseases, and Global Medicine, University of California, San Francisco, San Francisco, CA, United States; ^5^Department of Quantitative Health Sciences, John A. Burns School of Medicine, University of Hawaii at Manoa, Honolulu, HI, United States; ^6^Department of Tropical Medicine, Medical Microbiology and Pharmacology, John A. Burns School of Medicine, University of Hawaii at Manoa, Honolulu, HI, United States

**Keywords:** COVID-19, SARS-CoV-2, clinical presentation, epidemiology, risk factors, vaccines, treatments, public health

## Abstract

Coronavirus Disease 2019, caused by severe acute respiratory coronavirus 2, has been an ever-evolving disease and pandemic, profoundly impacting clinical care, drug treatments, and understanding. In response to this global health crisis, there has been an unprecedented increase in research exploring new and repurposed drugs and advancing available clinical interventions and treatments. Given the widespread interest in this topic, this review aims to provide a current summary—for interested professionals not specializing in COVID-19—of the clinical characteristics, recommended treatments, vaccines, prevention strategies, and epidemiology of COVID-19. The review also offers a historical perspective on the pandemic to enhance understanding.

## 1 Introduction

Coronavirus Disease 2019 (COVID-19), caused by severe acute respiratory syndrome coronavirus 2 (SARS-CoV-2), rapidly became a worldwide pandemic in 2020, leading to widespread illness and death. As the understanding of the disease and its impact evolves, and as the disease proceeds to endemicity, it is crucial to review and summarize the current knowledge of clinical features, symptoms, risk factors, epidemiology, treatments, vaccines, and prevention strategies. This review provides a comprehensive clinical overview of the current understanding of COVID-19.

## 2 Clinical features/symptoms and pathogenesis of COVID-19

**Figure 1:**
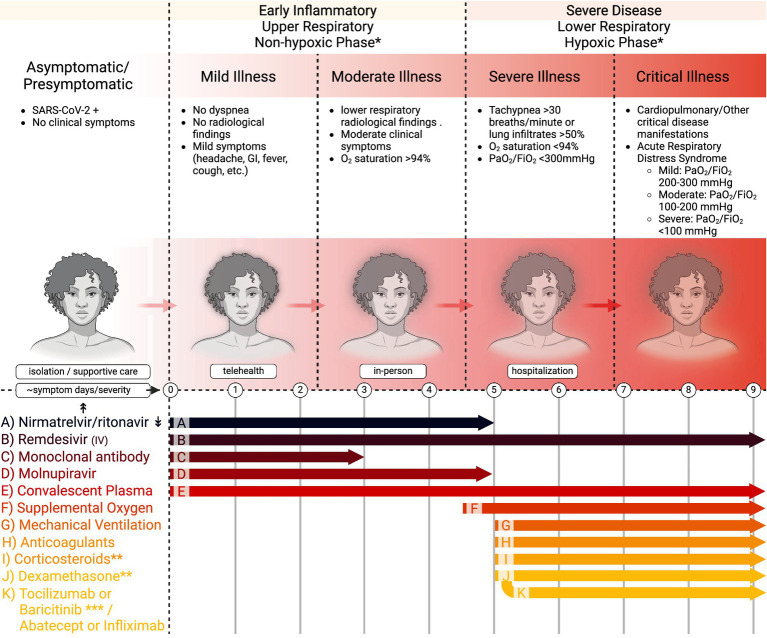
Stages of COVID-19 and recommended treatment timing in adults. This figure demonstrates the five stages of COVID-19, the corresponding findings associated with each stage, the recommended practice for physicians encountering patients in their respective stages (isolation, telehealth, in-person, and hospitalization), and the currently recommended treatment guidelines relating to time and severity of disease. *Care providers initiate based on contraindications and risk factors for more severe disease, and follow local protocols. **Final 2024 NIH Panel recommends against dexamethasone (I) or other corticosteroids (J) for COVID-19 treatment if no (F) or (G). *** If using dexamethasone + IV remdesivir, PO baricitinib or IV toxilizumab. If using dexamethasone, but not using or do not have access to IV remdesivir, IV adatacept or IV infliximab. ↟Dosing information can be obtained at dailymed.nlm.nih.gov ↡Drug–drug Interactions can be found at https://www.idsociety.org/paxlovid and https://www.covid19-druginteractions.org/checker. Further guidelines at UpToDate.com and www.covid19treatmentguidelines.nih.gov.

Whereas the clinical nature of the COVID-19 pandemic has evolved greatly following the roll-out of vaccines, updated vaccine boosters, and emergence and dominance of Omicron variants to a less morbid condition for many with dramatically lower hospital rates and virus-related deaths, moderate and severe acute disease is still observed with a mortality greater than influenza and other respiratory illnesses. The National Institutes of Health (NIH) classify five stages of COVID-19 based on severity (Figure 1) (1). These are asymptomatic or presymptomatic, mild, moderate, severe, and critical illnesses. The first stage, asymptomatic or presymptomatic, is when persons test positive for SARS-CoV-2 by a nucleic acid amplification test or antigen test but do not display clinical symptoms (1, 2). The mild illness stage is those patients without dyspnea or lower respiratory radiological findings but with other symptoms such as fever, cough, pharyngitis, malaise, cephalgia, nausea, or emesis. Those in the classification of moderate illness are persons with clinical symptoms, radiological findings of disease in the lower respiratory tract, and oxygen saturation > 94%. The severe illness stage is those with tachypnea at a respiratory frequency > 30 breaths/min, or lung infiltrates >50%, oxygen saturation < 94%, and partial pressure of oxygen/fraction of inspired oxygen (PaO2/FiO2) <300 mmHg. Critical illness is the most severe stage and includes patients who develop acute respiratory distress syndrome (ARDS) or display acute respiratory failure with septic shock or multiple organ dysfunction (1, 2). ARDS is a form of respiratory failure that requires clinical and radiological findings. ARDS progression is evaluated by decreasing PaO2/FiO2 levels from mild (200–300 mmHg) to moderate (100–200 mmHg) to severe (<100 mmHg).

**Figure 2:**
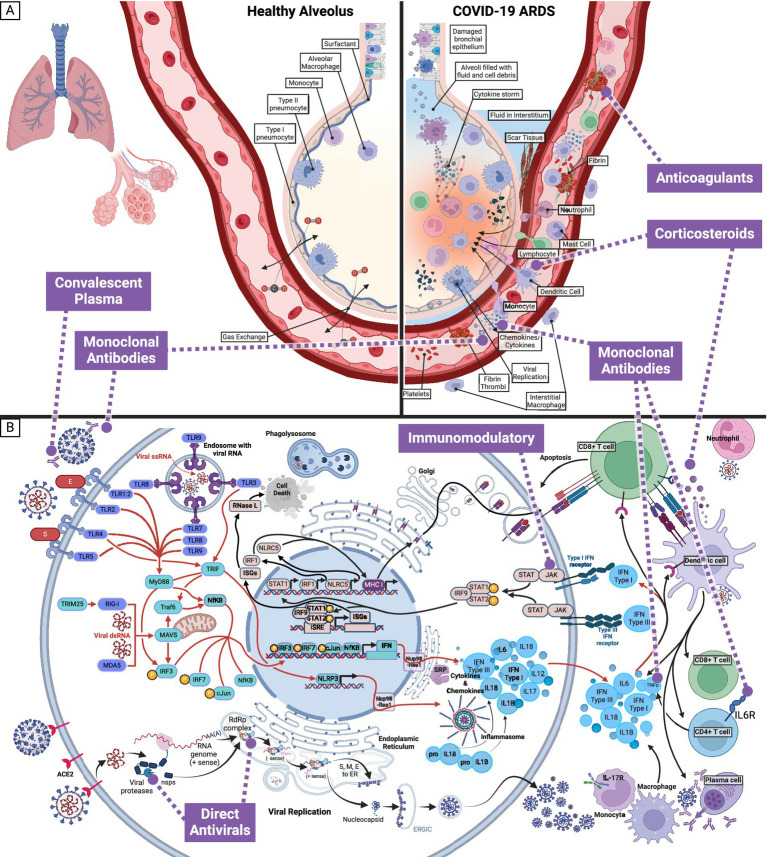
Effect of SARS-CoV-2 infection on healthy alveolus and pneumocyte, and associated targets of interventions. **(A)** In cases where SARS-CoV-2 infection progresses from the upper to the lower respiratory tract, the pathophysiological response can result in Acute Respiratory Distress Syndrome (ARDS). SARS-CoV-2 will preferentially target type II pneumocytes and cause infiltration by immune cells (macrophages, neutrophils, mast cells, and dendritic cells). The combined response of infected and immune cells will generate the cytokine storm and cause the endothelial cells to become leaky. The leaky endothelial cell junctions will leak fluid into the alveoli and interstitium, developing platelets and fibrin thrombi to compensate. The damaged cells, platelets, and thrombi will generate scar tissue. Together, these effects result in diffuse alveolar damage, which can be visualized histologically. The diffuse alveolar damage is the cause of the symptoms seen with critical COVID-19 illness and is known as Acute Respiratory Distress Syndrome. **(B)** Within a pneumocyte exposed to SARS-CoV-2, an intracellular inflammatory response and viral replication, lead to extracellular cytokines and inflammatory markers and infectious SARS-CoV-2. Medication categories (anticoagulants, convalescent plasma, corticosteroids, direct antivirals, immunomodulatory, and monoclonal antibodies) currently in use to target SARS-CoV-2 and alleviate the symptoms of COVID-19 are shown in purple.

ARDS (Figure 2A) is the hallmark of COVID-19 and accompanies a histological pattern known as diffuse alveolar damage (DAD). DAD includes edema, death of pneumocytes, thrombosis, capillary congestion, and hyaline membrane formation. The dead and dying pneumocytes will release cytokines and chemokines to recruit immune cells and cause inflammation (Figure 2B). Ultimately, the inflammatory response will damage microvascular endothelial cells, further causing leaky vessels. Hyaline membrane formation diminishes oxygen exchange, resulting from coagulation dysregulation and fibrotic signaling. Also, hyaline membranes will result in fibrin thrombi, depleting platelets and generating clots. Clotting further increases inflammation and is exacerbated by interleukin (IL)-6 production. The death of the lung epithelium and endothelium will result from viral replication, coagulation, and hypoxia and is the underlying pathology of pneumonia in SARS-CoV-2 infection (3, 4).

Symptomatic clinical presentations include dyspnea, fever, cough, pharyngitis, nausea, anorexia, anosmia, dysgeusia, cephalgia, malaise, myalgia, and diarrhea. Dyspnea, fever, and cough are the most common presentations in 70% of cases, followed by myalgia (36%) and cephalgia (34%) (2). SARS-CoV-2 infection, especially with pre-Omicron variants, can target the brain, eyes, nose, lungs, vasculature, liver, kidneys, and intestines. Approximately 23% of persons infected with SARS-CoV-2 will progress to severe COVID-19, with 5.6% of infected persons dying pre-Omicron and before widespread vaccination (5). Progression to more severe disease has become rare with the Omicron variants in those without risk factors. Symptoms relating to the gastrointestinal system, such as nausea and emesis, are associated with severe COVID-19 with pre-Omicron strains, as are symptoms of the respiratory system, such as angina and dyspnea (5). Finally, end-organ failure and pneumonia are associated with mortality (5).

## 3 Risk factors

Moving on to risk factors, risk factors for COVID-19 can be classified into environmental, viral, and host. Environmental factors include human crowding, occupational exposure, poor ventilation, and animal contact. On the other hand, viral risk factors are associated with the rapid evolution of SARS-CoV-2 throughout the pandemic and include transmissibility, evasive mutations, and viral loads associated with a particular variant (6).

Shifting our focus to host risk factors, most of which were identified prior to most persons experiencing vaccination or at least one infection, the primary host risk factors for COVID-19 in non-vaccinated individuals are old age, male sex, racial and ethnic minorities, diabetes mellitus, immunocompromised state, obesity, hypertension, lung disease, cardiovascular disease, cancer, pregnancy. For instance, advanced age is associated with COVID-19, intensive care unit (ICU) admission and mortality (6–11). This can be explained by the fact that age is associated with more comorbidities, weaker immune response, and septic shock complications that correlate with mortality (9, 12, 13). Moreover, males are more likely to acquire, be admitted to the ICU, and die from COVID-19 than females (5, 6, 8, 9). The underlying reasons for this sex difference include estrogen’s effect on solubilizing ACE2, levels of ACE2 and TMPRSS2, hormonal differences in the inflammatory response, health behaviors, personal concerns, social alarm, and responsible attitudes (6, 8, 9, 14, 15). Racial and ethnic minorities are also at higher risk for COVID-19 hospitalization and death. The reasons for this may include barriers to healthcare access, transportation, lack of insurance, and hesitancy about COVID-19 treatments (16). Other risk factors include diabetes mellitus, a known inflammatory disease shown to have immune system consequences (17). People with diabetes mellitus are at a higher risk for COVID-19, are less responsive to treatments, are more frequently admitted to the ICU, and are at higher risk of mortality (5–10, 13). An independent risk for people with diabetes mellitus is poorly controlled and elevated glucose (13, 18). Elevated glucose levels are also associated with increased ACE2 expression (9, 17, 18) and higher viral titers (19), as SARS-CoV-2 hijacks host cell metabolism (20, 21). Additionally, cardiovascular disease is a risk factor for COVID-19 due to the expression of ACE2 on cardiac myocytes and vascular fibroblasts (6, 7). Statin and aspirin use in diabetes and cardiovascular disease should be continued in those already taking them. Still, it should not otherwise be initiated during COVID-19 (22). Furthermore, immunodeficiency or immunosuppression increases the risk of severe disease and mortality in COVID-19 (23). Next, obesity in persons under 50 years of age increases the risk of hospitalization with COVID-19. Notably, obesity lengthened the stay of COVID-19 patients in hospitals (7, 9). As for hypertension, it causes a higher risk of acquiring COVID-19 and dying from the disease. Hypertension is related to the renin-angiotensin-aldosterone axis regulating blood pressure, (9, 24, 25), and a component of that axis is ACE2, the protein that SARS-CoV-2 binds to for entry. As many with hypertension take medications that decrease blood pressure, they will also be increasing ACE2 expression (6). However, the increase in mortality of hypertensives is related to the condition itself, and antihypertensives reduce the mortality in COVID-19 in those already on antihypertensive; thus, those on ACE inhibitors are often advised to continue to use them (22, 26). Lung diseases such as chronic obstructive pulmonary disease (COPD), interstitial lung disease (ILD), and pulmonary embolism are also risk factors for COVID-19 (9). Malignancy increases the risk of COVID-19, as it is associated with a weakened immune response, particularly when associated with chemotherapy (6). Pregnant women are more susceptible to COVID-19 infection than are non-pregnant women (9). Lastly, other host risk factors include malnutrition, autoimmunity, neurological disease, chronic kidney disease, smoking, and liver disease (6, 9–11).

## 4 Epidemiology

The SARS-CoV-2 virus first emerged in Wuhan, Hubei Province, China, on December 12, 2019 (27, 28). Upon its emergence, the Hubei Provincial Hospital notified the Chinese public health authorities that many unexplained pneumonia cases emerged from the Huanan market (29). Subsequently, the first report to the World Health Organization (WHO) of the outbreak of SARS-CoV-2 (then known as 2019-nCoV) was on December 31, 2019 (29–31). In a matter of weeks, by January 18, 2020, 2019-nCoV had spread to the United States, with the first reported case in Washington State (31, 32). Rapidly escalating, by February 12, 2020, more than 44,730 cases had been reported in China (28). Recognizing the severity, on March 11, 2020, the WHO declared the COVID-19 pandemic (30, 31, 33), and the White House announced on March 31, 2020, that 100,000–240,000 U.S. deaths were expected (31, 34). The Centers for Disease Control and Prevention (CDC) recommended facial masking guidelines in early April to curb the spread (31). In a further effort to contain the virus, 43 states of the United States had issued stay-at-home orders by April 24, 2020 (35). Despite these and other efforts, by November 30, 2020, SARS-CoV-2 had spread worldwide, infecting more than 62 million persons. Although vaccines gave hope to an end to the pandemic in early 2021, tragically, by early November 2021, there were more than 250 million confirmed cases and 5 million deaths worldwide. As of August 2024, that number had grown to 775 million cases and 7 million deaths worldwide, with over 103 million cases and 1.2 million deaths in the US (36, 37).

**Figure 3:**
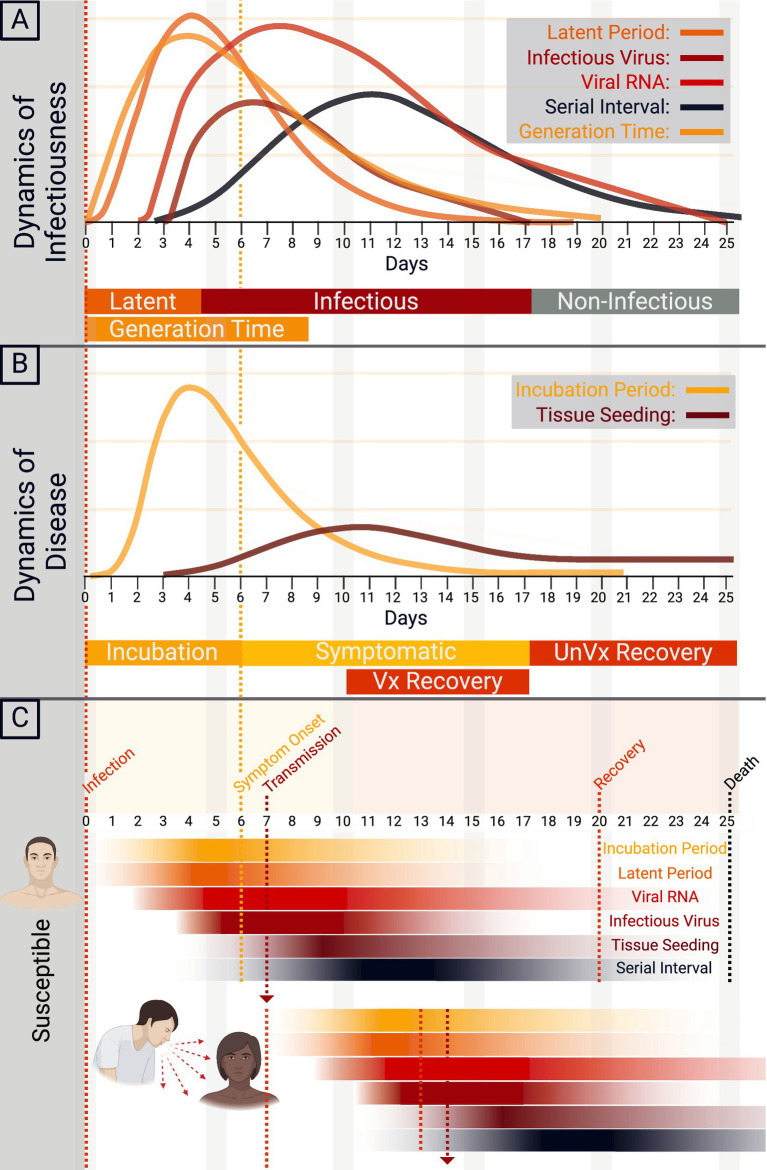
Natural history of COVID-19. **(A)** The dynamics of SARS-CoV-2 infectiousness are shown in A. The y-axis demonstrates a relative density for the latent period, infectious virus, viral RNA, serial interval, and generation time by time in days. **(B)** The dynamics of COVID-19 disease are shown in B, with the y-axis demonstrating a relative density of incubation period and tissue seeding by time in days. Approximate recovery time for vaccinated (Vx) and unvaccinated (UnVx) individuals is shown. **(C)** Schematic of the transmission of SARS-CoV-2 from primary case to secondary case, with the onset of symptoms and progression of disease.

The natural history of COVID-19 (Figures 3A–C), determined pre-Omicron, begins with exposure to SARS-CoV-2 (38–42). Upon exposure, the mean incubation period—the point of exposure to the onset of clinical signs—is between 5.8 and 6.9 days, ranging from 2.33 to 17.6 days. The range may vary due to age and infectious dose, and Omicron and other evolving strains will alter these metrics (38, 41, 43). As the infection develops, the latent period for SARS-CoV-2, the time between infection and infectiousness, is between 5.5 and 6.0 days (41, 44). Infectiousness and transmission start before symptom onset and peak at symptom onset (39). The intrinsic generation time—the interval between the infection dates of an infector and its secondary cases in a fully susceptible population—averages 6.84 days for the Omicron variant (45). The serial interval—the time between the onset of symptoms between successive cases—is between 4.8 to 6.8 days, with a mean of 5.8 days. During the infection, viral RNA load peaks near symptom onset or an average of 2 to 4 days post-infection and then gradually wanes, with infectiousness averaging 9.8 days post-symptom onset (38, 42). Interestingly, this waning corresponds with the approximate limit of detection of SARS-CoV-2 by viral RNA of 21 days (39). Immunocompromised persons, however, shed for much longer, with one study showing shedding for 151 days post-initial infection, during which time the virus evolved intra-host (46). IgG and IgM seroconversion occurs ~13 days following symptom onset (47). Some patients who cannot limit the infection to a mild illness will progress to severe disease. Severe disease can progressively be classified into the pulmonary phase and hyperinflammatory phase. The pulmonary phase of the disease happens an average of 5 days after symptom onset and is characterized by pneumonia and lung infiltrates (42). Unfortunately, some will further progress to the hyperinflammatory phase, characterized by ARDS discussed above (42). Ultimately, hospital discharge or death occurs at a mean of 18.1 days (15.1–21) from symptom onset (40). Tissue seeding is a concept that has come to light with the advent of Long COVID (48, 49). Tissue seeding likely begins during the initial viral infection and can be detected in organs throughout the body for weeks to years (49, 50). Major gaps in our knowledge of tissue seeding are currently being addressed. The viruses’ continued evolution and widespread vaccination will likely continue to alter these epidemiological characteristics.

## 5 Treatments and vaccines

The interventions in this pandemic are continuously evolving and involve vaccines and treatments, including small-molecule drugs, convalescent plasma, and monoclonal antibodies (summarized in Table 1 and Figure 2) (51, 52). As of 2024, the three recommended treatments for non-hospitalized COVID-19 in the United States are ritonavir-boosted nirmatrelvir (Paxlovid), remdesivir, and molnupiravir (53, 54). In contrast, for patients requiring hospitalization, nine treatments are presently in use depending on disease severity and therapeutic indications: remdesivir, dexamethasone, baricitinib, heparin, tofacitinib, tocilizumab, sarilumab, infliximab, and abatacept (54, 55). The European Medicines Agency refused marketing authorization for molnupiravir due to a lack of clinical benefit (56, 57). This decision followed the PANORAMIC study, which showed that molnupiravir did not reduce hospitalizations and death in a vaccinated population of high-risk adults during the omicron variant time period—and may contribute to further viral evolution (58).

**Table 1:** Current and historical FDA-approved and EUA drugs for COVID-19, including drugs commonly used off-label in COVID-19.

Type	Class	Names	Brief summary	FDA status COVID-19 (2024)
Monoclonal Antibody	Anti-IL6R (Immunomodulatory)	tocilizumab (Actemra)	IL6R; block inflammatory pathway to prevent disease progression	FDA approval
		sarilumab (Kevzara)	IL6R; block inflammatory pathway to prevent disease progression	off-label *
	Anti-TNFalpha (Immunomodulatory)	infliximab (Avsola, Flixabi, Inflectra, Remicade, Renflexis, Zymfentra)	TNFɑ; reduce key inflammatory cytokine, thereby reducing capillary leak	off-label *
	Anti-complement (Immunomodulatory)	vilobelimab (Gohibic)	complement factor 5a	FDA EUA
	Anti-SARS-CoV-2	casirivimab + imdevimab (REGN-COV2)	SARS-CoV-2 Spike glycoprotein	EUA revoked
		sotrovimab (Xevudy (VIR-7831))	SARS-CoV-2 Spike glycoprotein	EUA revoked
		bamlanivimab (LY-CoV555) ^^	SARS-CoV-2 Spike glycoprotein	EUA revoked
		etesevimab (LY-CoV016) ^^	SARS-CoV-2 Spike glycoprotein	EUA revoked
		tixagevimab + cilgavimab (EVUSHELD)	SARS-CoV-2 Spike glycoprotein	EUA revoked
		regdanvimab (Regkirona (CT-P59))	SARS-CoV-2 Spike glycoprotein	EUA revoked
		bebtelovimab (LY-CoV1404)	SARS-CoV-2 Spike glycoprotein	EUA revoked
		pemivibart (Pemgarda (VYD222))	pre-exposure prophylaxis of COVID-19; SARS-CoV-2 Spike glycoprotein	FDA EUA
Plasma		convalescent plasma	plasma with high titers of anti-SARS-CoV-2 antibodies	FDA EUA
Biologic		abatacept (Orencia)	fusion protein (CTLA4-Ig) disease-modifying anti-rhematic drug; selective T cell costimulation modulator	off-label *
Small molecule	Corticosteroids	dexamethasone	anti-inflammatory or immunosuppressant agent	off-label *
		hydrocortisone	glucocorticoid used to treat endocrine, immune, and allergic disorders	off-label *
		methylprednisolone	anti-inflammatory or immunosuppressive drug	off-label *
		prednisone (Deltasone, Rayos, Winpred)	anti-inflammatory or immunosuppressive drug	off-label *
	Immunomodulatory	baricitinib (Olumiant)	JAK inhibitor used to treat rheumatoid arthritis; believed to interfere with viral entry	FDA approval
		anakinra (Kineret)	recombinant antagonist of IL1R	FDA EUA
		tofacitinib (Xeljanz)	JAK inhibitor used to treat rheumatic conditions/ulcerative colitis/COVID-19	off-label *
	Direct Antivirals	molnupiravir (Lagevrio)	isopropylester cytidine analog; uptake by RdRp	FDA EUA
		nirmatrelvir + ritonavir (Paxlovid)	protease inhibitor + CYP 3A4 inhibitor	FDA approval
		remdesivir (Veklury)	nucleoside analog; binds RdRp	FDA approval
	Other	hydroxychloroquine (Plaquenil, Sovuna)	disease-modifying anti-rhematic drug	EUA revoked **
		chloroquine	antimalarial drug also used in rheumatoid arthritis	EUA revoked **
		heparin (Defencath, Heparin Leo)	anticoagulant; directly inhibit the conversion fibrinogen to fibrin by blocking the activity of factor IV and activating anti-factor 10 which neutralizes the effects	off-label *
		propofol-lipuro (Diprivan)	sedative to assist mechanical ventilation	EUA revoked
		propoven (Diprivan)	sedative to assist mechanical ventilation	EUA revoked
Other		multiFiltrate PRO System	CRRT	FDA EUA
		REGIOCIT replacement solution	replacement colution in CRRT	FDA EUA

Janus Kinase (JAK); Interleukin-6 Receptor (IL6R); Severe acute respirtory syndrome coronavirus 2 (SARS-CoV-2); Food and Drug Administration (FDA); Emergency Use Authorization (EUA); RNA-dependent RNA polymerase (RdRp); cytochrome P450 (CYP). *FDA approved for other indications. Off-label use in the setting of COVID-19. **Use is not indicated. Unclear clinical benefit. No rigorous prospective data showing efficacy. ^^eventually co-administered. FDA status current as of 04/2024.

### 5.1 Small molecules and approved drugs

Given the importance of small molecules in the treatment landscape, mainly those approved by the Food and Drug Administration (FDA) discussed below, it is essential to summarize their collection as shown in Table 1. As of 2024, the small molecules used to treat COVID-19 can be divided into four categories: corticosteroids, JAK inhibitors, direct antivirals, and others.

Even with the wide use of Emergency Use Authorization (EUA) and off-label, as shown in Table 1, there are only four drugs with complete FDA approval for treating COVID-19. The first FDA-approved drug for treating COVID-19 requiring hospitalization was remdesivir (Veklury) on October 22, 2020. Remdesivir is an SARS-CoV-2 RNA-dependent RNA polymerase inhibitor, essential for viral replication. Three randomized controlled trials contributed to its approval from manufacturer Gilead, including the ACTT-1 trial, which found that the median time to recovery with remdesivir was 10 days compared to 15 on placebo, a statistically significant difference (59). An open-label multicenter trial of hospitalized adults with moderate COVID-19 showed that the odds of a patient’s symptoms improving were higher in those who received 5 days of remdesivir versus placebo (60). The third study helped determine the optimal duration of treatment of 5 days as these patients had similar outcomes compared to those 10 days of therapy (61).

Baricitinib (Olumiant) was approved on May 10, 2022, for treating COVID-19 for hospitalized adults requiring supplemental oxygen, non-invasive or invasive mechanical ventilation, or extracorporeal membrane oxygenation. Manufactured by Eli Lilly, the proposed mechanism is inhibition of the JAK–STAT signaling pathway and inhibition of AP2-associated protein kinase, which controls viral endocytosis (62). Approval was based upon data published from two phase 3, randomized, double-blind, placebo-controlled clinical trials. The first showed an improvement in time to recovery when baricitinib was combined with remdesivir vs. placebo with remdesivir in adults hospitalized with COVID (63). The second trial demonstrated that fewer patients died or progressed to ventilation within 4 weeks when treated with baricitinib vs. placebo (64).

Next, to be approved by the FDA was tocilizumab (monoclonal antibody). Tocilizumab (Actemra) was approved for hospitalized adult patients receiving systemic corticosteroids requiring supplemental oxygen, non-invasive or invasive mechanical ventilation, or extracorporeal membrane oxygenation. Manufactured by Genentech, the drug selectively and competitively binds to the IL-6 receptor, theoretically reducing lung tissue injury caused by COVID-19 (65). Data compiled from several trials contributed to its approval on December 21st, 2022. In the RECOVERY trial, 4,116 hospitalized patients with severe COVID-19 pneumonia were randomized, and primary analysis revealed a statistically significant difference in the probability of death by day 28 in the tocilizumab group versus standard of care (66).

The final FDA-approved agent in the treatment of COVID-19 is nirmatrelvir + ritonavir (Paxlovid). Nirmatrelvir is a peptidomimetic inhibitor of SARS-CoV-2 3C-like protease, which prevents viral replication. Ritonavir, an HIV-1 protease inhibitor, inhibits the CYP3A-mediated metabolism of nirmatrelvir, increasing plasma concentrations of nirmatrelvir. The combination drug, manufactured by Pfizer, was officially approved by the FDA on May 25, 2023, though it has been widely used under EUA since December 2021. The combination drug was approved based upon outcomes from the EPIC-HR study, which showed an 86% reduction in risk of COVID-19-related hospitalization or death from any cause through Day 28 in patients who started treatment with Paxlovid within 5 days of symptoms onset as compared to placebo (67). The EPIC-SR also supported its approval, as it showed a numerical reduction in COVID-19-related hospitalizations or death in a sub-group of non-hospitalized adults with confirmed COVID-19 who had at least one risk factor for progression to severe disease and who were fully vaccinated (68).

### 5.2 Convalescent plasma

During COVID-19, as with the previous outbreaks of Severe Acute Respiratory Syndrome (SARS) and Middle East Respiratory Syndrome (MERS), convalescent plasma was a safe and effective tool in treatment and post-exposure prophylaxis (69). Drawing from a long history of success, using convalescent plasma or serum to counter infectious diseases has been successfully used for over a century (69, 70). In the early stages of the pandemic, the need for convalescent plasma was prevalent mainly in the first year, wherein the only available treatment for COVID-19 was convalescent plasma or sparingly-successful repurposed antivirals (69). At that time, convalescent plasma was used in ~10% of all worldwide infected persons in the COVID-19 pandemic’s first year (71). Fast forward to today, convalescent plasma continues to have FDA EUA in the United States for immunosuppressed persons (72, 73). However, the research and evidence remain divergent in the consensus on the efficacy of convalescent plasma, and efficacy could include factors such as time from infection to infusion, antibody titer, plasma quality, and co-administration with corticosteroids (70, 74, 75). Nonetheless, the use of convalescent plasma appears safe (76).

### 5.3 Monoclinal antibodies

Monoclonal antibodies (mAbs) were the initial pursuit of many companies worldwide (51, 77, 78). In the early stages, at one point, nine anti-SARS-CoV-2 mAbs and two anti-interleukin-6 receptor (IL6R) mAbs had received FDA EUA (51, 79–82). However, most anti-SARS-CoV-2 monoclonal antibodies had diminished efficacy against the evolving SARS-CoV-2 variant strains (77, 83–85). The loss of efficacy is due to the development of mAbs and the evolution of SARS-CoV-2. Most mAbs were designed against the proteins from ancestral sequences of the virus (27). Consequently, as the virus evolved, many changes in the Spike glycoprotein resulted in an inability of the monoclonal antibodies to recognize their epitope and neutralize the virus. Subsequently, all anti-SARS-CoV-2 monoclonal antibody treatments that had received FDA EUA have since been revoked due to lost efficacy (79, 81, 84–90). Remarkably, only tocilizumab received FDA approval for use in COVID-19 from all monoclonal antibodies once in use. More recently, in 2024, one anti-SARS-CoV-2 mAb, Pemgarda, has received FDA EUA for pre-exposure prophylaxis of COVID-19 (91, 92).

### 5.4 Vaccine development

**Figure 4:**
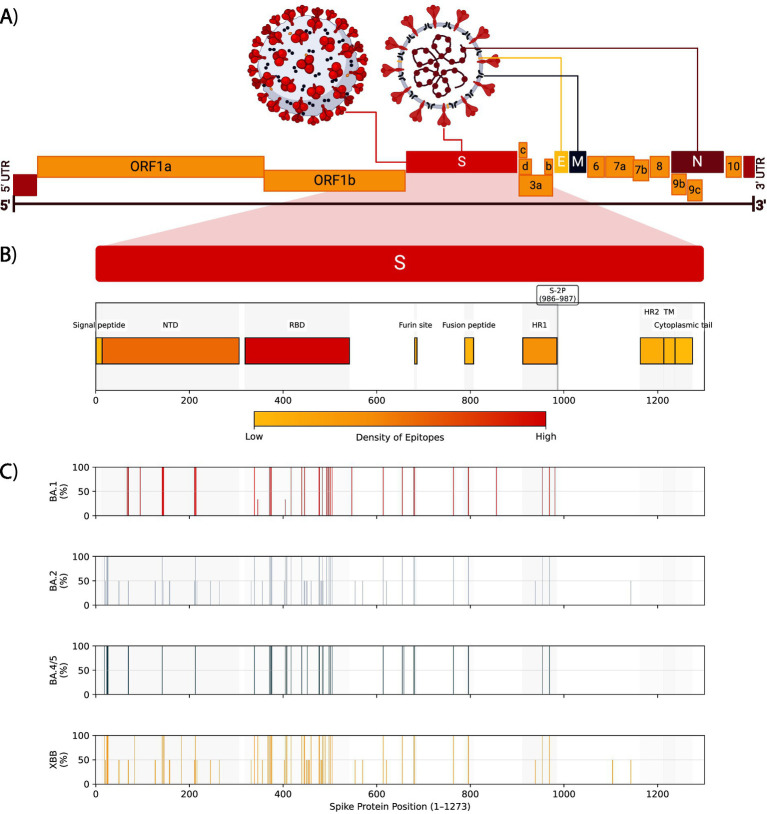
SARS-CoV-2 genome, spike protein domains, epitope densities, and omicron sublineage mutation frequencies. **(A)** The positive-sense RNA genome structure of SARS-CoV-2 showing the untranslated regions (UTR), structural proteins Spike (S), Envelope (E), Membrane (M), and Nucleocapsid (N), and non-structural proteins including ORF1a and ORF1b. **(B)** The Spike protein domains, including the signal peptide, N-terminal domain (NTD), receptor-binding domain (RBD), furin cleavage site, fusion peptide, heptad repeat 1 (HR1), HR2, transmembrane region (TM), and cytoplasmic tail. The S-2P pre-fusion stabilization site is shown at positions 986 and 987. The density of predicted B and T cell epitopes against pre-2022 variants (97) is shown as a color scale for each domain. **(C)** Bar plots showing mutation frequencies for BA.1, BA.2, BA.4/5, and XBB Omicron lineage families (98). Bar positions represent amino acid residue in Spike (1–1273), and the height represents the percentage of sublineages in that lineage family that contain a mutation at that residue.

Shortly after the publication of the first SARS-CoV-2 whole-genome sequences elucidated the ~30 kb genome (Figure 4A) (27, 93), the race to develop vaccines began, with the first in development by early 2020 (94, 95). Whereas traditional vaccine development has taken 15 years or longer, vaccine development to distribution with SARS-CoV-2 took between 10–17 months (95). These vaccines—including Pfizer and Moderna—were designed to elicit a response against the Spike glycoprotein (Figure 4B) (96). The Spike glycoprotein, one of four structural proteins in SARS-CoV-2, is critical for viral entry and antibody neutralization. Variants of concern have continuously evolved the Spike glycoprotein (Figure 4C), diminishing the efficacy of vaccines, monoclonal antibody treatments, and antiviral therapies. This ongoing evolution underscores the importance of continuous surveillance and research (58, 87, 97, 98).

Phase 1 trials for these vaccines began only 3 months into the pandemic, and by September 2020, there were hundreds in preclinical development, with many proceeding into clinical trials (95, 99). Many vaccine platforms were being tested, including live-attenuated, recombinant protein subunits, virus-like particles, replication-incompetent vectors, replication-competent vectors, inactivated virus, DNA, and RNA (95, 100–109). By early 2021, approximately 11 vaccines of five unique platform strategies made it through to phase 3 trials (108). These strategies and vaccines were:

1. RBD recombinant protein subunits.
2. Pre-fusion stabilized (S-2P) (110) lipid nanoparticle (LNP) mRNA.
3. S-2P replication-competent vectors.
4. Full-length S replication-competent virus vectors.
5. Whole inactivated virus (95).

Despite still being under clinical evaluation, the first SARS-CoV-2 vaccine received FDA EUA approval on December 11, 2020 (Pfizer and BioNTech BNT162b2) (94, 111). The BNT162b2 vaccine demonstrated an initial efficacy of 95% (94). BNT162b2, an S-2P LNP mRNA vaccine, became the first FDA-approved SARS-CoV-2 vaccine on August 23, 2021 (112, 113). Following suit, the S-2P LNP mRNA Moderna vaccine (mRNA-1273) also received full FDA approval on January 31, 2022 (114).

Other vaccines in the United States with FDA EUA approval included the S-2P Ad26 replication-incompetent vector Janssen vaccine (Ad26.COV2.S) and the S-2P protein subunit Novavax vaccine (NVX-CoV2373) (108, 115–117). The mRNA-1273, Ad26.COV2.S, and NVX-CoV2373 vaccines had initial efficacies of 94, 66, and 89.7%, respectively (117–120). Janssen later voluntarily withdrew their EUA. Globally, other vaccines against SARS-CoV-2 were approved worldwide by the WHO (95, 121). As of September 2021, 53 vaccines were being marketed and investigated for the future of SARS-CoV-2 vaccines, with 161 vaccine candidates by July 2022 (122, 123). Fast forward to March 2023, 382 SARS-CoV-2 vaccines were in pre-clinical or clinical development (124).

Initially, vaccines received approval as one or two doses for adults and have since progressed to include many age groups and boosters to three and four doses as the pandemic continues and the virus evolves (125–131). Additionally, as the SARS-CoV-2 virus is predicted to remain endemic (132), these vaccines will involve changing the sequences of the genomes and proteins as part of next-generation vaccine design to match the evolution of SARS-CoV-2, akin to influenza virus vaccines (97). Such has been demonstrated with the bivalent vaccines with the addition of Omicron strain (BA.4 and BA.5) spike proteins to the original Pfizer and Moderna vaccines (133).

The vaccination schedule and strategies are also an essential topic for consideration. While the Pfizer and Moderna vaccines initially recommended three and four-week intervals, respectively, (134) scheduling guidelines have since been updated to 3–8 weeks and 4–8 weeks, respectively (135). Currently, 8 weeks is the recommendation for persons who are not immunocompromised (136). Interestingly, the longer dosing interval also reduces the occurrence of COVID-19 vaccine-related myocarditis, which is highest among young males receiving mRNA vaccines (136–138). Moreover, research has shown that neutralizing antibody concentrations are up to 2.3-fold greater at 6–14 weeks (139). Initially, this extension of interval practice began in Europe as a method to increase the population with first-dose protection by delaying second doses. Still, researchers found the method effective and immunogenic, potentially reducing mortality (139, 140). A model predicting the optimal delay between the first and second dose elucidates 12 weeks as the optimal time (141).

Given the evolving nature of the virus, there are many factors to consider when evaluating vaccines against SARS-CoV-2, including variant effects, humoral and cellular responses, epitopes, delivery methods, and adjuvants. Firstly, SARS-CoV-2 Variants of Concern (VOC) do not compromise T cell responses. However, B cell responses and neutralizing antibodies significantly decrease against emerging VOC (142). As a result, future vaccines may also need to address presenting multiple epitopes beyond the Spike protein, as N and M-specific T-cell responses dominate in non-hospitalized and mild cases. Interestingly, in contrast, spike-specific T-cell responses are associated with more severe infection (143). Moving forward, when designing next-generation SARS-CoV-2 vaccines, many more factors must be considered. These considerations include dosing schedule, antigen presentation, and immunization route (144, 145). Nevertheless, regardless of possible improvements, the United States vaccination program is estimated to have prevented more than 235,000 deaths in the first 10 months (Dec 2020–Sep 2021) (146). On a global scale, this estimate increased to 19.8 million deaths prevented due to SARS-CoV-2 vaccines by December 8, 2021 (147).

## 6 Prevention

**Figure 5:**
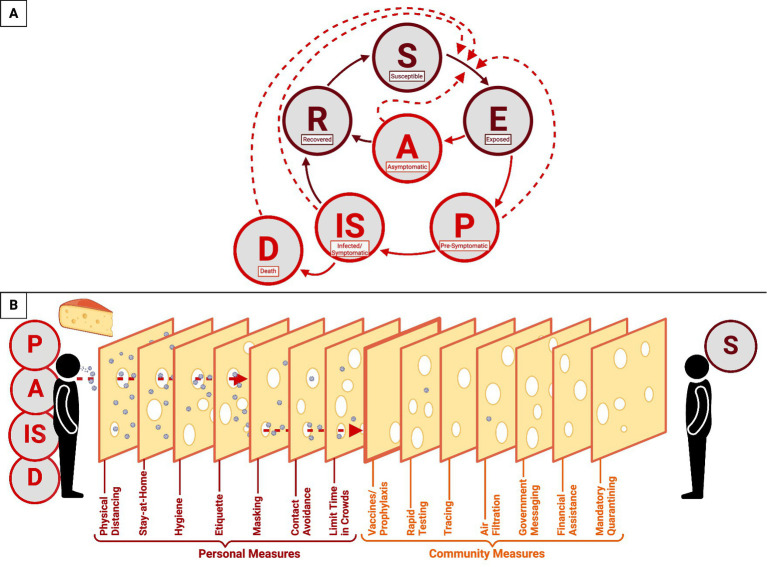
COVID-19 epidemiological model of SARS-CoV-2 transmission and prevention. This figure represents a combination of epidemiological models used to demonstrate the progression of the COVID-19 disease and the transmission of SARS-CoV-2. **(A)** The transmission cycle begins with a susceptible individual (S) who is exposed to the virus by a pre-symptomatic (P), asymptomatic (A), infected/symptomatic (IS), or deceased (D) individual. Once infected, the susceptible individual (S) will progress to the exposed (E) stage and become either asymptomatic or pre-symptomatic. If pre-symptomatic, the individual will proceed to the infected/symptomatic stage and will either recover (R) or die (D). If asymptomatic, the individual will recover and become recovered (R). Recovered individuals may re-enter the cycle as susceptible (S). Solid lines represent the stages of COVID-19 progression, while dashed lines represent the transmission of SARS-CoV-2. **(B)** The Swiss Cheese Model of pandemic defense exemplifies the response to the rapid spread of SARS-CoV2. This model includes both personal (crimson) and communal (orange) measures to limit the spread of the virus.

SARS-CoV-2 is transmitted by asymptomatic, pre-symptomatic, and symptomatically infected individuals (148). The transmission of SARS-CoV-2 proceeds from susceptible individuals to exposed individuals; exposed individuals can either remain asymptomatic and recover or become presymptomatic and infected. The infection then results in either recovery or mortality (Figure 5A) (148). Pre-symptomatic persons transmit 40–60% of new infections, and asymptomatic persons transmit <10% (148). This presymptomatic transmission is demonstrated by the median time between infection and symptom onset being 5.7–7 days (43, 94, 149). Furthermore, between 17.9 and 33.3% of patients infected with SARS-CoV-2 will remain asymptomatic (2). Otherwise, high transmission occurs ~2.5 days before symptom onset (148), which means that people who have not sought medical care or a diagnostic test can still transmit the virus. So much so that even vaccinated persons can shed and transmit the virus, which has been particularly prevalent with variants (113, 150–153). SARS-CoV-2 has a variable reproductive number (R0) from 0.52–5.08 that changes with new variant strains (154–156). The secondary attack rate was 16.6% in late 2020 but escalated to 19.4% with the Delta VOC and 25.1% with the Omicron VOC (157, 158). The case fatality rate began at 3.71% in March 2020 but decreased to 1.13% by July 2022 and is higher in areas with a low vaccination rate (155, 159).

Containment strategies were also implemented, including government-issued stay-at-home orders and travel restrictions. The pandemic defense was centered around overlapping responses that generate multiple layers of protection—known as the Swiss cheese model (Figure 5B) (160). This comprehensive model relies upon personal and communal measures to prevent the spread and transmission of the virus. Individual defense measures include physical distancing, staying at home, hygiene, etiquette, mask-wearing, contact avoidance, and limiting time in crowds. In parallel, communal measures include rapid testing, tracing, air filtration, government messaging, financial assistance, mandatory quarantining, and vaccines. Together, these responses served as the pandemic defense and public policy response for nearly two and a half years until the CDC lifted restrictions on masks in early 2022 (161).

## 7 Conclusion

The rapid spread of COVID-19 worldwide has vastly changed hospitals and treatments since the pandemic began in late 2019. This dynamic included many drugs and treatments once in use that have since been revoked due to ineffectiveness and a fluid dynamic facilitated by the evolving variants of SARS-CoV-2. This review aims to provide a complete summary of the present status of COVID-19. This collection signifies historical and present status in a constantly changing and active field.

The subjects covered herein must be continually reassessed and reflected upon as further evidence emerges globally. Reassessing and reflecting on these topics can improve pandemic response plans, refine treatment plans, and develop a more robust vaccine policy. To maintain the highest level of care, current treatments need to be monitored for maintained effectiveness, and treatment successes of other countries should be explored. To exemplify this, understanding how patients react during hospitalization can point to the success of hypnotics as used in China (12). By evaluating the current pandemic response plans, we can allow for response implementation sooner in future COVID-19 waves or other pandemics. Such a response will include masking, distancing, stay-at-home orders, and other components of the Swiss-cheese model. To develop a more robust vaccine policy and design future SARS-CoV-2 vaccines, we must continually monitor the viral evolution and incorporate the viral changes into our vaccines to maintain effectiveness and identify the correlates of protection necessary for an appropriate and long-lasting response. This vaccine evaluation should include evaluating multiple SARS-CoV-2 proteins in the vaccines and adjuvants that can produce the response needed for long-term protection and identifying algorithms for determining the emergence of variants such as flu vaccines (97). The persistent evaluation of these aspects facilitates optimal control and preparedness for COVID-19 and other potential pandemics.
